# Eating behaviors, dietary patterns and weight status in emerging adulthood and longitudinal associations with eating behaviors in early childhood

**DOI:** 10.1186/s12966-022-01376-z

**Published:** 2022-11-16

**Authors:** Lise Dubois, Brigitte Bédard, Danick Goulet, Denis Prud’homme, Richard E. Tremblay, Michel Boivin

**Affiliations:** 1 School of Epidemiology and Public Health, University of Ottawa, 600 Peter Morand, Ottawa, ON K1G 5Z3 Canada; 2 Université de Moncton, Campus de Moncton, Pavillon Léopold-Taillon, 18, avenue Antonine-Maillet, Moncton, NB E1A 3E9 Canada; 3 Research Unit on Children’s Psychosocial Maladjustment (GRIP), Université de Montréal, 3050, Édouard-Montpetit, Montréal, QC H3T 1J7 Canada; 4 UCD School of Public Health, Physiotherapy and Sports Science, University College Dublin, Belfield, Dublin 4, Ireland; 5 École de psychologie, Université Laval, 2325 allée des Bibliothèques, Québec, QC G1V 0A6 Canada

**Keywords:** Eating behavior, Diet, Weight, Longitudinal study, Children, Adults, Appetitive traits, Food acceptance

## Abstract

**Background:**

Eating behaviors may contribute to differences in body weight and diet over time. Our study aims to examine how eating behaviors of young adults relate to their current weight status and dietary patterns and to explore longitudinal associations with eating behaviors in early childhood.

**Methods:**

Study participants are young adults (*n* = 698) taking part in the Quebec Longitudinal Study of Child Development. At age 22, eating behaviors were assessed using the Adult Eating Behavior Questionnaire. Dietary patterns were derived from information collected by food frequency questions. Weight status was based on self-reported data. Information on eating behaviors in childhood had been collected when participants were 2.5 to 6 years old. Pearson’s correlations were used to determine associations between adult eating behaviors and body mass index. Simple and multivariate linear regression analyses were used to examine associations between eating behaviors and dietary patterns at age 22, and longitudinal associations with behaviors in early childhood. Ordinal logistic regression analyses were used to assess associations between overeating and fussy eating in childhood and weight status at age 22.

**Results:**

Body mass index was positively correlated with Emotional overeating, Enjoyment of food, and Food responsiveness and negatively correlated with Satiety responsiveness, Emotional undereating, Slowness in eating and Hunger. A Healthy dietary pattern was positively associated with both Enjoyment of food and Hunger, and negatively associated with Food fussiness. Inversely, a Beverage-rich dietary pattern was negatively associated with Enjoyment of food and positively associated with Food fussiness. A Protein-rich pattern was positively associated with Enjoyment of food, while a High energy density pattern was positively associated with Food fussiness. Young adults with higher scores for fussy eating in early childhood were more likely to manifest Food fussiness and Emotional undereating, and less likely to adopt a Healthy dietary pattern. Young adults with higher scores for overeating in early childhood were less likely to show traits such as Slowness in eating and more likely to be overweight.

**Conclusions:**

Our findings suggest that eating behaviors in childhood have long-term influence on diet and weight status, thereby reinforcing the importance of early interventions that promote healthy eating.

**Supplementary Information:**

The online version contains supplementary material available at 10.1186/s12966-022-01376-z.

## Background

Diet is recognized as one of the key modifiable factors for obesity prevention [1]. In developed countries such as Canada, many young people now reach adulthood overweight or obese [2], a condition that puts them at higher risk for premature death from chronic disease [3, 4]. During “emerging adulthood” (ages 18 to 25) [5], most young adults transition from high school to college or university, as well as to part- or full-time work. They leave their parents’ homes to live alone, with friends, or as couples; some start families [5]. This developmental stage requires that they learn how to choose, buy, and prepare food and meals daily, activities that can pose dietary and nutritional challenges [5, 6]. Indeed, a variety of studies have confirmed that this transition period coincides with declines in diet quality‚ accompanied by rapid changes in weight [5–7].

When compared to childhood and adolescence, health-enhancing behaviors (e.g., diet, physical activity) in emerging adulthood has not received the attention they deserve by public health research [4]. This oversight is critical because emerging adulthood offers opportunities for interventions targeting excess weight and, more broadly, for obesity prevention [5, 6]. Indeed, as young adults construct their personal, familial, and social identities, they typically become more receptive than older adults to adopting lifelong, healthy lifestyles [5]. Preventive interventions at this age are also timely because a large proportion of these young adults are‚ or will eventually become parents who are likely to pass their dietary habits on to the next generation.

Designing evidence-based preventive interventions among young adults would benefit from a keen understanding of eating behaviors that contribute to body weight and dietary habits over time. To some extent, these eating behaviors reflect family attitudes and customs, as well as tastes and preferences developed earlier in life [8, 9]. They may also be influenced by genetic factors underpinning food intake regulation and taste predispositions [10, 11].

In childhood, selective eating behaviors (picky/fussy eating) have been associated with reduced enjoyment of food, smaller meals, slow eating, higher liquid intakes, and more limited dietary variety [12, 13], while overeating and fast eating have been associated with higher body mass index (BMI) [14, 15]. Previous research by our team indicated that preschool-aged children who were perceived as fussy by their mothers tended to under-consume certain types of food, such as fruit, vegetables, and meat (and alternatives) [16]. Young children perceived as eating too much or too fast had higher energy intake and higher BMI [15]. Such eating behaviors tend to persist throughout childhood [17, 18], although evidence for continued persistence into adulthood remains limited [9, 19].

Identifying how past and current eating behaviors relates to diet and weight status among young adults would deepen our understanding of the behavioral aspects of diet. Accordingly, the first objective of this study was to examine the extent to which eating behaviors of young adults relate to their current weight status and dietary patterns. A secondary objective was to document the predictive associations between eating behaviors in early childhood, as reported by parents, and both eating habits (i.e., eating behaviors and dietary patterns) and weight status in early adulthood.

## Method

### Participants

Study participants were young adults taking part in the ongoing Quebec Longitudinal Study of Child Development (QLSCD). Under the direction of the Institut de la Statistique du Québec (ISQ) [20], the QLSCD is a birth cohort study that was initiated in the late 1990s. It was intended to deepen understanding of interactions between various factors influencing children’s psychosocial and cognitive development. A total of 2120 children (51% boys) entered the cohort in 1998 at 5 months of age. The children of this cohort constituted a representative sample of more than 75,000 singleton births in Quebec that year. Details about the QLSCD are available elsewhere [21]. Twenty-two years later, 700 participants agreed to take part in a dietary study in which information on food consumption, eating behaviors, anthropometric data, and sociodemographic factors was collected using an online self-administered questionnaire. Data collection took place in the spring of 2020 (from March through June inclusively). Because two participants had missing data for the eating behavior questionnaire, the study sample includes 698 young adults, all of whom were 22 years of age. Comparisons between our sample and other QLSCD participants in the original cohort indicated that our sample included a larger proportion of women (65% vs 41%, *p* < 0.001; see Supplementary Table 1). Our sample also had a lower proportion of participants born to families of lower socioeconomic status (relative to household income and maternal education, *p* < 0.001) or whose mothers were younger (*p* = 0.044) or immigrants (*p* = 0.005).

### Eating behaviors

Eating behaviors at age 22 were assessed using the Adult Eating Behavior Questionnaire (AEBQ) [22]. The AEBQ, recently developed and based on the well-known Child Eating Behavior Questionnaire (CEBQ) [23], assesses a broad array of eating traits related to appetite and food acceptance. More specifically, this questionnaire includes 35 items measuring 8 eating traits. These refer to four food approach scales (Hunger [5 items], Food responsiveness [4 items], Emotional overeating [5 items], Enjoyment of food [3 items]) and four food avoidance scales (Satiety responsiveness [4 items], Emotional undereating [5 items], Food fussiness [5 items], Slowness in eating [4 items]) [22]. The AEBQ has been translated into several languages, including French, and was found to be a valid and reliable instrument for assessing eating traits in different adult populations [22, 24–28]. In published validation studies, various factor structures of the AEBQ have been compared. Although some studies have suggested that a 7-factor structure excluding the Hunger scale might be appropriate [25, 26, 28], the validation study conducted in Quebec supported the original 8-factor structure of the questionnaire [24], which was used in the present study. For each item, a score (from 1 to 5) was derived from responses on a 5-point Likert scale (Strongly disagree, Disagree, Neither agree nor disagree, Agree, Strongly agree). For each participant, the scores of all items related to a given trait were summed and then divided by the number of items for that trait in order to obtain a mean score for each of the 8 eating traits.

In earlier rounds of the QLSCD study, specific eating behaviors have been documented at five points in time (i.e., at ages 2.5, 3.5, 4.5, 5, and 6 years) through interview with the “most knowledgeable person” about the child (the mother, for the most part). Questions were based on those used in the Avon Longitudinal Study of Parents and Children [29], then translated and slightly adjusted to reflect the context of the QLSCD. Five questions were of particular interest for the present study: 1*) When [name of the child] is at home with you for the main meal of the day, how often does he/she eat a meal that differs from meals that other family members eat? In general, does [name of the child]…* 2) *…refuse to eat the right food?* 3) *…refuse to eat?* 4) *… over-eat?* 5) *…eat too fast?* Possible answers included “Almost never (1 point), Sometimes (2 points), Almost always (3 points), Always (4 points)”, for the first question, and “Never (1 point), Rarely (2 points), Sometimes (3 points), Often (4 points)” for the last four questions. For each participant, at each data collection point between age 2.5 and 6 years, the sum of the scores for “fussy eating” (range: 3–12) was calculated from responses to the first three questions (“eating different meals”, “refusing to eat the right food”, and “refusing to eat”). Similarly, the sum of the scores for “overeating” (range: 2–8) was calculated from responses to the last two questions (“overeating” and “eating too fast”). For both “fussy eating” and “overeating”, a mean score for the whole period (between 2.5 and 6 years) was then derived by considering all years for which a participant had complete data for that behavior. It is worth mentioning that all participants had data available at least once over the five data collection rounds.

### Food consumption at age 22 years

We assessed frequency of consumption of 60 food/food-group items, covering a large array of dietary sources in the diet of Canadian adults. Participants were asked to think about a typical week (or 7-day period, before the COVID-period that was just beginning at the time) and to indicate if they were consuming each food item listed. If so, they were asked to indicate how often (whether by day or by week) and in what quantities (based on three common portion sizes). Answers specifying frequencies per week were converted into frequencies per day to yield a common frequency unit. For smaller, average and larger suggested portions sizes (specific to each food item), we assigned factors of 0.5, 1.0 or 1.5, respectively. Relative quantities of the 60 food items consumed were determined by multiplying frequency per day by portion size factor. Each food item was then assigned to one of the following 16 food groups: 1) Sugar-sweetened beverages (SSB); 2) Milk and plant-based drinks (unsweetened); 3) Juice; 4) Fruit; 5) Vegetables; 6) Whole-grain products; 7) Non whole-grain products; 8) Processed meat (including pizza and fried poultry/fish/shellfish); 9) Red meat; 10) Poultry/fish/shellfish (excluding fried items) & eggs; 11) Legumes, nuts & seeds; 12) Cheese; 13) Yogurt; 14) Fatty/salty snacks (including French fries); 15) Sweet snacks and desserts; 16) Alcohol. Relative quantities of each food group were determined by calculating the sum of relative quantities of individual food items included in a given group.

### Anthropometric data and other covariates

Information on current weight and height at the time of the dietary study was reported by participants and used to calculate BMI (weight [kg]/height[m]^2^). Correction equations, based on Canadian data for adult men and women of various age groups, were applied to adjust self-reported data in order to improve accuracy relative to measured data [30]. Weight status was determined based on World Health Organization BMI classification: Underweight (BMI < 18.5); Normal weight (BMI 18.5–24.9); Overweight (BMI 25.0–29.9); Obese (BMI ≥30.0). Because two participants had missing data for BMI, analyses that included BMI refer to 696 participants.

Sex of the participant, maternal education, and family income had already been collected in earlier rounds of the QLSCD study and were used as covariates in longitudinal analyses. As part of the dietary study at age 22, participants were questioned about their usual living situation (e.g., living alone or with a partner, friends, parents) and the highest level of education or training they had taken courses in. Sex, living situation, level of education, and weight status of the participants were used as covariates in analyses relating eating behaviors and food consumption at age 22 years.

### Statistical analysis

We used exploratory factor analysis to derive dietary patterns that summarize how various food groups combine to characterize various types of food consumption in our sample. Since consumption of individual food groups did not follow a normal distribution, the principal axis method of estimation, which does not require distribution assumptions, was used. Parallel analysis scree plots suggested the optimal number of factors to be 4. Goodness of fit across models with 2 to 5 factors was compared using fit indices such as the Tucker-Lewis index (TLI; value > 0.95 indicates good fit); the Root Mean Square Error of Approximation (RMSEA; value ≤0.5 indicates good fit); and the Bayesian Information Criterion (BIC). This analysis confirmed that a 4-factor solution best described eating patterns in our sample. Factor loading estimation for the 4 groups was performed using principal axis factoring with Oblimin rotation to identify the model that best explained the interrelationships among these food groups. Model fit was satisfactory with a TLI of 0.86 and a RMSEA of 0.042. Using other factor loading estimation methods (minimum residuals, ordinary least squares, unweighted least squares) did not affect individual factor loadings and fit measures. Factor loadings ≥0.3 were considered.

Descriptive statistics included frequency and mean (SD). Bivariate (Pearson’s) correlations were used to determine associations between AEBQ scales, between AEBQ scales and BMI, and between AEBQ scales and the consumption of specific food groups. Comparisons of AEBQ mean scores, by sex and weight status categories, were performed using one-way ANOVA. For Enjoyment of food and Food fussiness, we used Spearman correlations and the Kruskal-Wallis Rank Sum Test because these variables were not normally distributed. Tukey multiple comparisons of means were used for factory variables having more than 2 categories when differences were observed. Simple linear regressions analyses were used to test whether eating behavior traits measured by the AEBQ were associated with dietary patterns. When a significant association (*p* < 0.05) was detected, adjusted models (for sex, education, living situation, and weight status at age 22) were tested.

As part of a longitudinal analysis, we used linear regressions to explore bivariate associations between eating behaviors in childhood (mean scores for overeating and fussy eating) and eating habits (i.e., eating behaviors and dietary patterns) in emerging adulthood. When a significant association was detected (*p* < 0.05), we tested adjusted models controlling for sex, maternal education, and family income. We also assessed whether eating behaviors in childhood were predictors of weight status at age 22. Since this outcome variable comprises three categories (underweight/normal weight; overweight; obese), we used ordinal logistic regression which assumes proportional odds between each pair of categories to be compared (i.e., the effect of a predictor is constant from categories 1 to 2 and 2 to 3 of the outcome). We tested both crude and adjusted (for sex, maternal education, and family income) models. In a sensitivity analysis, we also explored the possible moderating role of sex on the longitudinal analysis by adding an interaction term between sex and the predictor (“fussy eating” or “overeating”) to the adjusted models. We performed all analyses by using RStudio [31] with R Statistical Software [32] version 4.1.2. Significance level was set at 0.05.

## Results

As shown in Table 1, our study sample included a majority of women (65%). Among all participants, more than half (55%) had already enrolled in university courses, 48% were still living with one or two parents at the time of the dietary study, and a quarter (25%) were living with a partner, with or without children. Of those participating, 44% were overweight or obese.

**Table 1:** Characteristics of young adult participants (*n* = 698)

Characteristics	% (n) or mean ± SD
Sex	
Male	34.8 (243)
Female	65.2 (455)
Age	22.2 ± 0.25
Highest level of education (courses)	
Secondary school or less	18.9 (132)
College studies	25.8 (180)
University studies	55.3 (386)
Living situation	
Living alone	7.7 (54)
Living with parents	47.9 (334)
Living with a partner, with/without children	25.1 (175)
Other situation (incl. combination)	19.3 (135)
BMI^a^	25.8 ± 6.0
Weight status^b^	
Underweight	1.9 (13)
Normal weight	53.9 (376)
Overweight	26.9 (188)
Obese	17.0 (119)
Missing	0.3 (2)
Eating behaviors in early childhood	
Fussy eating^c^	5.79 ± 1.39
Overeating^d^	2.80 [2.20, 3.40]

*BMI* Body mass index

^a^ BMI was calculated as kg/m^2^ based on self-reported height and weight - corrected [30]

^b^ Based on World Health Organization classification of BMI: Underweight (BMI < 18.5), Normal weight (BMI 18.5–24.9); Overweight (BMI 25.0–29.9); Obese (BMI ≥30.0)

^c^ Range: 3–12

^d^ Range: 2–8. Overeating presented as median [IQR] since the distribution is not normally distributed

Bivariate correlations between AEBQ scales are shown in Table 2. Positive correlations were noted among all food approach scales (from *r* 0.16, *p* < 0.01, between Enjoyment of food and Hunger, to *r* 0.50, *p* < 0.01, between Enjoyment of food and Food responsiveness). Food avoidance scales also showed positive correlations with one another (from *r* 0.20, *p* < 0.01, between Slowness in eating and Emotional undereating, to *r* 0.37, *p* < 0.01, between Slowness in eating and Satiety responsiveness), except in the case of Food fussiness (where correlation was limited to Satiety responsiveness; *r* 0.15, *p* < 0.01). Food approach scales were mostly negatively correlated with food avoidance scales (from *r* − 0.09, *p* < 0.05, between Food responsiveness and Slowness in eating, to *r* − 0.24, *p* < 0.01, between Enjoyment of food and Food fussiness). Hunger was one exception, given that this trait showed positive correlations with food avoidance scales (from *r* 0.09, *p* < 0.05, in relation to Satiety responsiveness, to *r* 0.18, *p* < 0.01, in relation to Emotional undereating, although not statistically significant in relation to Food fussiness [*r* 0.04]).

**Table 2:** Bivariate (Pearson’s) correlations between AEBQ scales and between AEBQ scales and BMI

AEBQ scales	H	FR	EOE	EF	SR	EUE	FF	SE	BMI^a^
Food approach									
Hunger (H)	1.00								−0.076*
Food responsiveness (FR)	0.368**	1.00							0.080*
Emotional overeating (EOE)	0.191**	0.350**	1.00						0.253**
Enjoyment of food (EF)	0.156**	0.500**	0.197**	1.00					0.095*^b^
Food avoidance									
Satiety responsiveness (SR)	0.090*	−0.168**	−0.131**	−0.224**	1.00				−0.144**
Emotional undereating (EUE)	0.182**	−0.027	−0.234**	−0.093*	0.330**	1.00			−0.087*
Food fussiness (FF)	0.037	−0.095*	0.054	−0.237**	0.146**	0.031	1.00		0.057^b^
Slowness in eating (SE)	0.092*	−0.088*	−0.069	−0.136**	0.365**	0.196**	−0.001	1.00	−0.098**

*AEBQ* Adult Eating Behavior Questionnaire, *BMI* Body mass index

^a^ BMI was calculated as kg/m^2^ based on self-reported height and weight - corrected [30]. Missing values for BMI (n = 2)

^b^ For *Enjoyment of food* and *Food fussiness*, *r* values refer to Spearman correlations because these variables were not normally distributed

n = 698; *p < 0.05; **p < 0.01

Table 2 also presents results of bivariate correlation analyses between BMI and each eating trait. Overall, BMI was positively correlated with three food approach scales (Emotional overeating [*r* 0.25, *p* < 0.001], Enjoyment of food [*r* 0.095, *p* = 0.013], and Food responsiveness [*r* 0.08, *p* = 0.036]). Inversely, BMI was negatively correlated with all food avoidance scales (from *r* − 0.09, *p* = 0.021 for Emotional undereating, to *r* − 0.14, *p* < 0.001 for Satiety responsiveness) except Food fussiness. Hunger, contrary to the other food approach scales, was also found to be negatively correlated with BMI, but modestly so (*r* − 0.08, *p* = 0.045).

As shown in Table 3, food approach scales tend to have higher mean scores (mean ± SD varying between 2.51 ± 0.96 and 4.33 ± 0.66) compared to food avoidance scales (varying from 2.02 ± 0.84 to 2.95 ± 0.98). For all appetitive traits but one (Food fussiness, which does not differ according to sex), the mean score is higher for women than for men. Differences according to weight status were also noted, namely for two food approach scales and three food avoidance scales. Among the food approach scales, we noted an upward gradient in mean score for Emotional overeating, from normal/underweight to overweight and to obese categories. The mean score for Enjoyment of food is also higher for overweight compared to normal/underweight categories. Inversely, for three food avoidance scales, we noted higher mean scores among normal/underweight individuals compared to those having excess weight (i.e., overweight or obese individuals; for Satiety responsiveness and Slowness in eating) or compared to obese individuals only (for Emotional undereating).

**Table 3:** AEBQ mean scores for all participants, by sex and by weight status

AEBQ scales		Sex			Weight status^a^			
	All (n = 698)	Men (*n* = 243)	Women (*n* = 455)		Normal/ Underweight (*n* = 389)	Overweight (*n* = 188)	Obese (*n* = 119)	
	Mean ± SD	Mean ± SD	Mean ± SD	*P* value^b^	Mean ± SD	Mean ± SD	Mean ± SD	*P* value^b^
Food approach								
Hunger	2.80 ± 0.72	2.67 ± 0.66	2.88 ± 0.75	< 0.001	2.83 ± 0.75	2.81 ± 0.69	2.70 ± 0.69	0.192
Food responsiveness	3.26 ± 0.72	3.15 ± 0.70	3.32 ± 0.72	0.003	3.21 ± 0.70	3.34 ± 0.73	3.30 ± 0.76	0.104
Emotional overeating	2.51 ± 0.96	2.34 ± 0.92	2.60 ± 0.97	0.001	2.29 ± 0.88^c^	2.67 ± 0.95^d^	2.98 ± 1.02^e^	< 0.001
Enjoyment of food	4.33 ± 0.66	4.22 ± 0.67	4.38 ± 0.65	0.001	4.25 ± 0.71^c^	4.47 ± 0.56^d^	4.34 ± 0.61	0.003
Food avoidance								
Satiety responsiveness	2.69 ± 0.77	2.39 ± 0.65	2.85 ± 0.78	< 0.001	2.78 ± 0.82^c^	2.61 ± 0.69^d^	2.55 ± 0.70^d^	0.004
Emotional undereating	2.95 ± 0.98	2.70 ± 0.97	3.08 ± 0.96	< 0.001	3.04 ± 1.02^c^	2.89 ± 0.93	2.77 ± 0.93^d^	0.020
Food fussiness	2.02 ± 0.84	2.00 ± 0.83	2.03 ± 0.85	0.808	1.97 ± 0.84	2.03 ± 0.85	2.13 ± 0.82	0.081
Slowness in eating	2.67 ± 1.00	2.35 ± 0.96	2.84 ± 0.98	< 0.001	2.79 ± 0.97^c^	2.49 ± 1.05^d^	2.55 ± 0.96^d^	0.001

*AEBQ* Adult Eating Behavior Questionnaire, *BMI* Body mass index

^a^ Based on World Health Organization classification of BMI: Underweight (BMI < 18.5), Normal weight (BMI 18.5–24.9); Overweight (BMI 25.0–29.9); Obese (BMI ≥30.0). BMI was calculated as kg/m^2^ based on self-reported height and weight - corrected [30]. Few participants were classified as underweight (< 2% of the sample) and thus were grouped with the normal weight category. Missing values for weight status (*n* = 2)

^b^ *P* value from one-way ANOVA. For *Enjoyment of food* and *Food fussiness*, *p* value refers to Kruskal-Wallis Rank Sum Test because these variables were not normally distributed

^c-e^ For a given trait, different letters indicate a significant difference (*p* < 0.05) between two weight-status categories based on Tukey multiple comparisons of means

Exploratory factor analysis suggested four dietary patterns that we identified as Healthy, Beverage-rich, Protein-rich, and High energy density (Table 4). Results of bivariate correlations between appetitive traits and consumption of specific food groups are presented as Supplementary material. (See Supplementary Table 2 for correlations with food approach scales and Supplementary Table 3 for correlations with food avoidance scales.)

**Table 4:** Food groups associated with dietary patterns identified and factor loadings

Dietary Patterns	Food groups	Factor loadings
Healthy	Legumes, nuts & seeds	0.64
	Whole-grain products	0.60
	Vegetables	0.52
	Fruit (excluding juice)	0.46
Beverage-rich	Sugar-sweetened beverages	0.51
	Milk & plant-based drinks (unsweetened)	0.40
High energy density	Processed meat (including pizza & fried poultry/fish/shellfish)	0.47
	Alcohol	0.43
	Cheese	0.38
	Fatty/salty snacks (including French fries)	0.33
Protein-rich	Red meat	0.49
	Poultry/fish/shellfish (excluding fried) & eggs	0.42

Based on exploratory factor analysis. Extraction method: principal axis factoring; Rotation method: Direct Oblimin; Cutting value of extracting factors: 0.3. Other food groups not included in dietary patterns: Sweet snacks and desserts; Juice; Non whole-grain products; Yogurt

Table 5 presents statistically significant associations between AEBQ scales and dietary patterns in emerging adulthood, based on findings from simple regression analyses presented in Supplementary Table 4. Multiple regression analyses indicated that models including Food fussiness accounted for a large proportion of variance in most dietary patterns (Healthy: 17%, R^2^_adj_ = 0.174; Beverage-rich: 16%, R^2^_adj_ = 0.161; High energy density: 16%, R^2^_adj_ = 0.156). A Healthy dietary pattern was positively associated with both Enjoyment of food (ß_adj_ = 0.14, SE = 0.05, *p* = 0.005) and Hunger (ß_adj_ = 0.09, SE = 0.04, *p* = 0.042), in addition to being negatively associated with Food fussiness (ß_adj_ = − 0.33, SE = 0.04, *p* < 0.001). Inversely, a Beverage-rich dietary pattern was negatively associated with Enjoyment of food (ß_adj_ = − 0.11, SE = 0.04, *p* = 0.01) and positively associated with Food fussiness (ß_adj_ = 0.18, SE = 0.03, *p* < 0.001). The Protein-rich pattern was positively associated with Enjoyment of food (ß_adj_ = 0.12, SE = 0.04, *p* = 0.003), while the High energy density pattern was positively associated with Food fussiness (ß_adj_ = 0.10, SE = 0.03, *p* = 0.002). Both the Protein-rich and the High energy density patterns were found to be negatively associated with Satiety responsiveness in unadjusted models, but these associations disappeared in adjusted models.

**Table 5:** Adult Eating Behavior Questionnaire (AEBQ) scales associated with dietary patterns at age 22 years

Dietary pattern	Unadjusted (n = 698)			Adjusted^a^ (*n* = 696)				
AEBQ scale	ß	(SE)	*P* value	ß	(SE)	*P* value	R^2^	R^2^_adj_
Healthy								
Hunger	0.108*	(0.044)	0.014	0.088*	(0.043)	0.042	0.084	0.072
Enjoyment of food	0.164**	(0.048)	0.001	0.136**	(0.048)	0.005	0.089	0.077
Food fussiness	−0.355**	(0.035)	< 0.001	− 0.329**	(0.035)	< 0.001	0.184	0.174
Beverage-rich								
Enjoyment of food	−0.167**	(0.042)	< 0.001	−0.106**	(0.041)	0.010	0.140	0.129
Food fussiness	0.205**	(0.032)	< 0.001	0.178**	(0.031)	< 0.001	0.171	0.161
Protein-rich								
Enjoyment of food	0.084*	(0.041)	0.043	0.122**	(0.041)	0.003	0.096	0.084
Satiety responsiveness	−0.105**	(0.035)	0.003	−0.039	(0.036)	0.283	0.086	0.074
High energy density								
Satiety responsiveness	−0.100**	(0.035)	0.006	−0.015	(0.035)	0.669	0.155	0.144
Food fussiness	0.106**	(0.032)	0.002	0.095**	(0.031)	0.002	0.167	0.156

Based on linear regressions testing whether appetitive traits are associated with dietary patterns

*R*^*2*^_*adj*_ Adjusted R squared

^a^Analyses adjusted for sex, education, living situation, and weight status of participants at age 22 years

*p < 0.05; **p < 0.01

Longitudinal multiple regression analyses indicated that fussy eating in early childhood positively predicted later Food fussiness (ß_adj_ = 0.14, SE = 0.02, *p* < 0.001) and Emotional undereating (ß_adj_ = 0.06, SE = 0.03, *p* = 0.03) and negatively predicted a Healthy dietary pattern (ß_adj_ = − 0.05, SE = 0.02, *p* = 0.04) at 22 years (Table 6; see also Supplementary Table 5 for bivariate associations between eating behaviors in early childhood and eating behaviors/patterns at age 22 years). Overeating in early childhood negatively predicted Slowness in eating (ß_adj_ = − 0.14, SE = 0.04, *p* < 0.001) in early adulthood. Although overeating in childhood was found to be positively associated with Satiety responsiveness and with the Beverage-rich pattern in crude models, these predictive associations disappeared in adjusted models. Finally, children having higher scores for overeating in their early years appeared more likely to be overweight or obese in emerging adulthood (OR_adj_ = 1.44; 95% CI: 1.23–1.70; *p* < 0.001; see Supplementary Table 6 for associations between eating behaviors in early childhood and weight status at age 22 years). There was no statistically significant association between fussy eating in childhood and weight status at 22 years.

**Table 6:** Eating behaviors in early childhood associated with eating behaviors and dietary patterns at age 22 years

Predictor	Unadjusted (n = 698)			Adjusted^a^ (*n* = 693)				
Outcome	ß	(SE)	*P* value	ß	(SE)	*P* value	R^2^	R^2^_adj_
Fussy eating in early childhood								
Emotional undereating	0.06*	(0.03)	0.04	0.06*	(0.03)	0.03	0.06	0.05
Food fussiness	0.14**	(0.02)	< 0.001	0.14**	(0.02)	< 0.001	0.07	0.07
Healthy dietary pattern	−0.05*	(0.02)	0.05	−0.05*	(0.02)	0.04	0.08	0.07
Overeating in early childhood								
Satiety responsiveness	−0.07*	(0.03)	0.03	−0.06	(0.03)	0.07	0.09	0.08
Slowness in eating	−0.15**	(0.04)	< 0.001	−0.14*	(0.04)	< 0.001	0.08	0.07
Beverage-rich dietary pattern	0.07*	(0.03)	0.03	0.03	(0.03)	0.36	0.14	0.13

Based on linear regressions testing whether scores for eating behaviors in early childhood are predictors of appetitive traits or dietary patterns at age 22 years

*QLSCD* Quebec Longitudinal Study of Child Development, *R*^*2*^_*adj*_ Adjusted R squared

^a^ Analyses adjusted for sex and both maternal education and family income when QLSCD participants were children

*p < 0.05; **p < 0.01

We detected four significant sex interactions (see Supplementary Table 7). Stratified analyses showed a significant association exclusively in women between fussy eating in childhood on the one hand, and both Food responsiveness (Female: ß = 0.05, 95% CI: 0.00–0.10; *p* = 0.047) and Emotional overeating (Female: ß = 0.08, 95% CI: 0.01–0.14; *p* = 0.016) at 22 years, on the other hand. Additionally, fussy eating in childhood was negatively associated with Enjoyment of food in men (ß = – 0.07, 95% CI: − 0.13–0.02; *p* = 0.011), but not in women.

## Discussion

The present study used data from a large population-based birth cohort to document how eating behaviors relate to dietary patterns and body weight in young adulthood and then, how certain eating behaviors in early childhood predict these eating traits and patterns‚ as well as weight status. Our findings indicate that among young adults, eating traits such as Emotional overeating, Enjoyment of food, and Food responsiveness were positively associated with BMI. Inversely, traits such as Hunger, Emotional undereating, Satiety responsiveness, and Slowness in eating were negatively associated with BMI. Food fussiness, Enjoyment of food, and Hunger were associated with certain dietary patterns after adjusting for various potentially confounding factors. Over time, we noted that young adults with higher scores for fussy eating in early childhood were more likely to manifest Food fussiness and Emotional undereating, and less likely to adopt a Healthy dietary pattern. Young adults with higher scores for overeating in early childhood were less likely to show traits such as Slowness in eating and more likely to be overweight.

Overall, the results relative to AEBQ and body weight are consistent with previous studies [22, 24–26], suggesting a general tendency toward higher mean scores in food approach scales and lower mean scores in food avoidance scales as BMI increases [22, 25, 26] (or in higher compared to lower weight status categories [24]). Hunger, which was inversely associated with BMI, and Food Fussiness, which was not associated with BMI or weight status, were two exceptions that had previously been reported in various AEBQ validation studies [22, 25]. Enjoyment of food and Hunger were the only two traits related to both dietary patterns and body weight (BMI and/or weight status). Food fussiness was related only to dietary patterns.

To our knowledge, this is the first study linking AEBQ appetitive traits and food consumption. Some experts have suggested that Food fussiness could reflect tastes and preferences more than appetite [24, 25]. Accordingly in the present study, Food fussiness was associated with dietary patterns that gave more prominence to beverages and high-energy foods, including SSB, processed meats, fried foods, cheese, and alcohol. Moreover, Food fussiness was also associated with lower consumption of healthy foods, such as vegetables and fruit, whole-grain products, legumes, nuts, and seeds. The few studies on food fussiness and picky eating among adults appear to be in line with our findings, suggesting a tendency toward lower-quality diets among selective eaters (also known as fussy or picky eaters) [19], particularly when lower consumption levels of vegetables and fruit, and less diversity in food choices, are considered [33, 34]. Our results also suggest that processed foods high in sugar, salt, or fat (or any combination of those) hold more appeal for those who have greater sensitivity to particular flavors.

In the present study, unlike Food fussiness, Enjoyment of food was associated with higher-quality diets. Besides being positively associated with a Healthy dietary pattern and negatively associated with a Beverage-rich pattern, Enjoyment of food was also positively related to dietary patterns rich in animal protein (including red meat, poultry, fish/shellfish, and eggs). Thus, those who show more pronounced interests in food and enjoy eating may appreciate a variety of nutrient-dense foods. Interestingly, a study of cultural differences in food perceptions in France versus the United States indicates that Americans tend to associate unhealthy food with tastiness and gustatory pleasure, whereas in France healthy food is perceived as tastier and more gratifying [35].

This cultural difference may, in part, explain our findings. Our mainly French-speaking North American population maintains strong cultural connections with other francophone cultures. However, the present study has also revealed that scores for Enjoyment of food among the overweight participants are higher than for the normal weight/underweight participants. This suggests that, beyond their interest in the quality of food, individuals who enjoy eating may also have, overall, higher energy intakes relative to energy requirements. It is worth mentioning that differences in Enjoyment of food relative to weight status did not apply to the obese category. Thus, beyond a certain point, excess weight and its potential consequences for health and psychosocial issues may instead be associated with restrictions in dietary intakes (i.e., to control or to lose weight), thus limiting eating-associated pleasures.

Hunger was another trait positively associated with a healthy dietary pattern, but only in relation to consumption of legumes, nuts, and seeds. When vegetarianism was considered in the equation (from information collected in the dietary study), the association between Hunger and Healthy dietary pattern vanished. It may be that Hunger is specifically related to characteristics of vegetarian diets (where legumes, nuts, and seeds represent a major source of protein), and not to healthy eating in general. Hunger was also positively correlated with sweets snacks and desserts, suggesting that there might be a propensity to rely on sources of free sugars in response to feelings of hunger.

Overall, the inverse relationship between Hunger and BMI may indicate that higher scores on the Hunger scale reflect lower energy intakes associated with greater awareness of internal hunger signals. Accordingly, a study among adult women (*n* = 1601) in New Zealand indicated that being attentive to hunger and satiety signals, which characterize intuitive eating, is related to lower BMI [36]. Interestingly, our results show a positive correlation between Hunger and Satiety responsiveness. We also found a negative correlation between Satiety responsiveness and BMI.

The other appetitive traits measured in our study were related to weight status, but not to any specific dietary pattern, suggesting that these traits contribute more to quantities of food ingested (total energy intake) than to types (quality) of food consumed. These traits include Emotional overeating and undereating, Food responsiveness, Satiety responsiveness, and Slowness in eating. Indeed, a tendency to overeat or to eat rapidly, a high responsiveness to food cues, or a lack of attention to satiety signals may all lead to excess energy intakes (relative to energy needs), and ultimately to excess weight [37].

Findings of our longitudinal analyses suggest that eating behaviors related to regulation of appetite or food acceptance in the younger years tend to emerge early in life and may persist until adulthood. Although there is no comparable study using AEBQ, a study conducted among UK children (*n* = 322) has looked at the stability and continuity of similar constructs measured by the CEBQ between ages 4 to 11 [17]. The authors of the study concluded that several eating traits may be as stable over time as personality traits are. Interestingly, we have found associations in early adulthood that were similar to our earlier research, when QLSCD participants were children. For example, being fussy about food was, at the time, related to lower intakes of vegetables and fruit and of meat and alternatives [16]; in young adulthood, Food fussiness was indeed inversely associated with the consumption of specific food groups such as Vegetables, Fruit, Poultry, fish and eggs, and Legumes, nuts and seeds. Likewise, children perceived by their parents as often eating too much or too fast were, at the time, more likely to have higher energy intakes and higher BMI compared to others [15]. In adulthood, higher scores for Emotional overeating and Food responsiveness, and lower scores for Slowness in Eating and Satiety responsiveness were associated with a higher BMI. Young adults with excess weight were also more likely to be perceived as overeating when they were children.

Other research findings at a very young age (i.e., from 16 months to 3–4 years) also appear in line with our observations in young adulthood. For example, a study among British (*n* = 1044) and Australian (*n* = 167) children using the CEBQ (from which AEBQ was derived) reported that liking vegetables and liking fruits were both positively associated with Enjoyment of food and negatively associated with Food fussiness [38]. The findings that parents readily identified problematic eating behaviors in their children indicate that early interventions, starting with the preschool years, may have long-term benefits. This highlights the importance of providing the knowledge and tools that parents and caregivers need to assist children with tendencies toward persistent food fussiness or overeating, whether at home or in a day care center, in order to improve long-term diet quality and to promote healthy weight. At the public health level, policies and programs would also benefit from considering behavioral aspects that promote healthy eating through childhood to young adulthood.

To the best of our knowledge, our cohort study is the first to use the AEBQ to study eating traits in young adulthood in relation to food intakes and to examine, via longitudinal analysis, how some of these traits may be related to behaviors at early ages. The 8-factor structure of the AEBQ has already been validated for the adult population living in the Canadian province of Quebec [24]. Our study relied on a relatively large sample of young adults similar in age. They had been followed since birth as part of a large birth cohort study that was representative of children born in 1998 in Quebec. Still, due to attrition over the years, our sample differs from the original cohort in certain sociodemographic characteristics (e.g., overrepresentation of women, underrepresentation of participants from lower socio-economic groups). For that reason, our findings may underestimate some associations given the restricted sample distribution; it may also not be generalizable to young adult populations living in Quebec today.

Food intakes were estimated using food-frequency questions covering a large number of food items. Identification of dietary patterns also allowed considering how various types of food are consumed in combination. Still, all dietary assessment methods are prone to measurement errors. For example, consumption of certain types of food may have been subject to social desirability bias. BMI and weight status were assessed through self-reported anthropometric measures, which is a limitation. Nevertheless, accuracy was improved by correcting these values based on equations developed and published for our population. The present findings are also consistent with other published studies. Even though the effect size of some correlations or associations remains low to moderate, depending on the outcome, the overall picture that emerges from our findings, and particularly the direction of associations, points to meaningful results from both a research perspective and a public-health perspective.

Finally, although the assessments of eating behaviors in childhood and in emerging adulthood did not rely on the same psychometric instrument, we were able to explore comparisons over time having to do with behaviors related to both appetite and food acceptance. Still, further longitudinal studies based on the CEBQ and the AEBQ, both of which refer to similar constructs, might be of considerable interest and utility in assessing the long-term continuity of specific eating traits. It is worth mentioning that by using a mean score to characterize eating behaviors in childhood, we may have overlooked some nuances when it comes to variations from one time to another. Nevertheless, we found moderate-to-high correlations between periods, which suggests a certain stability of these behaviors over time.

## Conclusions

The present study found several associations between eating behaviors measured by the AEBQ and both body weight and food consumption in emerging adulthood. Investigating and understanding eating behaviors among young adults thus may prove helpful in designing adaptive strategies in intervention programs aiming to promote healthy eating and healthy weight in this population group. Our findings also support the idea that eating behaviors take root early in childhood, thereby reinforcing the need for early interventions that have the potential to alter intergenerational obesity cycles while promoting nutritional health over the life course.

## Supplementary Information

**Additional file 1: Supplementary Table 1.** Comparison of participant characteristics between study sample and other participants in original QLSCD cohort.

**Additional file 2: Supplementary Table 2.** Bivariate (Pearson's) correlations between food approach scales of AEBQ and consumption of various food groups.

**Additional file 3: Supplementary Table 3.** Bivariate (Pearson's) correlations between food avoidance scales of AEBQ and consumption of various food groups.

**Additional file 4: Supplementary Table 4.** Bivariate associations between AEBQ scales and dietary patterns at age 22 years.

**Additional file 5: Supplementary Table 5.** Bivariate associations between eating behaviors in early childhood and eating behaviors/patterns at age 22 years.

**Additional file 6: Supplementary Table 6.** Associations between eating behaviors in early childhood and weight status at age 22 years.

**Additional file 7: Supplementary Table 7.** Significant sex interactions in longitudinal analyses and stratified effects of eating behaviors in early childhood.

## Data Availability

The data that support the findings of this study are available from ISQ but restrictions apply to the availability of these data, and so are not publicly available. Data are however available from the corresponding author (LD) upon reasonable request and with permission of ISQ.

## Abbreviations

AEBQ: Adult Eating Behavior Questionnaire
BMI: Body mass index
CEBQ: Child Eating Behavior Questionnaire
ISQ: Institut de la Statistique du Québec
QLSCD: Quebec Longitudinal Study of Child Development
RSMA: Root Mean Square Error of Approximation
SSB: Sugar-sweetened beverages
TLI: Tuckey Lewis Index
