# Supplementary material for: Eating behaviors, dietary patterns and weight status in emerging adulthood and longitudinal associations with eating behaviors in early childhood
Source: Int J Behav Nutr Phys Act. 2022 Nov 16;19:139. doi: 10.1186/s12966-022-01376-z (PMC9670577; doi:10.1186/s12966-022-01376-z)
Supplement: Supplementary file 1 — Additional file 1: Supplementary Table 1. Comparison of participant characteristics between study sample and other participants in original QLSCD cohort. [file 12966_2022_1376_MOESM1_ESM.docx]

**Supplementary Table 1** Comparison of participant characteristics between study sample and other participants in original QLSCD cohort

| Characteristics ^a^ | % (n) | | *P* value |
| --- | --- | --- | --- |
|  | Study sample  (n=698) | Other participants in original QLSCD cohort  (n=1422) |  |
| Sex |  |  |  |
| Male | 34.8 (243) | 58.9 (837) | <0.001 |
| Female | 65.2 (455) | 41.1 (585) |  |
| Birth weight |  |  |  |
| < 2500 g | 3.5 (24) | 3.2 (44) | 0.769 |
| 2500-4000 | 85.0 (588) | 86.1 (1200) |  |
| > 4000 g | 11.6 (80) | 10.7 (149) |  |
| Preterm birth |  |  |  |
| Yes | 5.3 (37) | 4.4 (62) | 0.392 |
| No | 94.7 (661) | 95.6 (1360) |  |
| First-born child in family |  |  |  |
| Yes | 45.8 (320) | 43.3 (616) | 0.292 |
| No | 54.2 (378) | 56.7 (806) |  |
| Maternal age |  |  |  |
| < 20 y | 1.6 (11) | 3.4 (48) | 0.044 |
| 20-34y | 84.0 (586) | 83.7 (1189) |  |
| 35+ y | 14.5 (101) | 12.9 (184) |  |
| Maternal education |  |  |  |
| < Secondary school diploma | 15.2 (106) | 19.6 (279) | <0.001 |
| Secondary school diploma | 22.4 (156) | 28.1 (399) |  |
| Post-sec. except university | 29.0 (202) | 28.8 (409) |  |
| University diploma | 33.4 (233) | 23.5 (333) |  |
| Annual household income (CAD) |  |  |  |
| < 30 000 $ | 22.7 (157) | 33.5 (465) | <0.001 |
| 30 000 - < 60 000$ | 43.4 (301) | 39.0 (542) |  |
| 60 000 - <80 000$ | 18.8 (130) | 14.2 (197) |  |
| ≥ 80 000 $ | 15.2 (105) | 13.3 (185) |  |
| Sufficient income |  |  |  |
| Yes | 82.3 (575) | 72.1 (1034) | <0.001 |
| No | 17.7 (123) | 27.9 (388) |  |
| Maternal immigrant status |  |  |  |
| Born in Canada | 91.0 (635) | 86.6 (1232) | 0.005 |
| Immigrant | 9.0 (63) | 13.4 (190) |  |

QLSCD, Quebec Longitudinal Study of Child Development; CAD, Canadian Dollar.

^a^ Information collected when QLSCD participant was aged 5 months. Perinatal data obtained from birth records.
