# Supplementary material for: Eating behaviors, dietary patterns and weight status in emerging adulthood and longitudinal associations with eating behaviors in early childhood
Source: Int J Behav Nutr Phys Act. 2022 Nov 16;19:139. doi: 10.1186/s12966-022-01376-z (PMC9670577; doi:10.1186/s12966-022-01376-z)
Supplement: Supplementary file 2 — Additional file 2: Supplementary Table 2. Bivariate (Pearson's) correlations between food approach scales of AEBQ and consumption of various food groups. [file 12966_2022_1376_MOESM2_ESM.docx]

**Supplementary Table 2** Bivariate (Pearson’s) correlations between food approach scales of AEBQ and consumption of various food groups

|  | Hunger | |  | Food responsiveness | |  | Emotional overeating | |  | Enjoyment of food^a^ | |
| --- | --- | --- | --- | --- | --- | --- | --- | --- | --- | --- | --- |
|  | Corr | *P* value |  | Corr | *P* value |  | Corr | *P* value |  | Corr | *P* value |
| Sugar-sweetened beverages | 0.040 | 0.295 |  | −0.034 | 0.375 |  | 0.004 | 0.916 |  | −0.167** | <0.001 |
| Fatty/salty snacks & French fries | 0.013 | 0.734 |  | 0.028 | 0.455 |  | 0.015 | 0.696 |  | −0.036 | 0.342 |
| Sweet snacks & desserts | 0.082* | 0.029 |  | 0.059 | 0.120 |  | 0.061 | 0.106 |  | 0.024 | 0.522 |
| Fruit | 0.020 | 0.601 |  | −0.003 | 0.945 |  | −0.003 | 0.943 |  | 0.141** | <0.001 |
| Juice | 0.001 | 0.985 |  | −0.054 | 0.153 |  | −0.093* | 0.014 |  | −0.004 | 0.919 |
| Vegetables | 0.026 | 0.486 |  | −0.008 | 0.829 |  | −0.001 | 0.988 |  | 0.171** | <0.001 |
| Non-whole-grain products | 0.044 | 0.244 |  | 0.088* | 0.020 |  | 0.034 | 0.363 |  | 0.014 | 0.721 |
| Whole-grain products | 0.051 | 0.177 |  | −0.049 | 0.195 |  | 0.071 | 0.062 |  | 0.036 | 0.338 |
| Processed meat, pizza & fried  chicken/fish/shellfish | −0.028 | 0.459 |  | −0.053 | 0.162 |  | −0.070 | 0.065 |  | −0.064 | 0.094 |
| Red meat | −0.041 | 0.279 |  | −0.031 | 0.420 |  | −0.054 | 0.154 |  | −0.042 | 0.268 |
| Poultry, fish, shellfish (excluding fried) & eggs | 0.031 | 0.416 |  | −0.003 | 0.929 |  | 0.022 | 0.562 |  | 0.109** | 0.004 |
| Legumes, nuts & seeds | 0.135** | <0.001 |  | 0.073 | 0.054 |  | 0.060 | 0.111 |  | 0.098** | 0.009 |
| Milk & plant-based drinks (unsweetened) | −0.015 | 0.695 |  | −0.041 | 0.279 |  | −0.031 | 0.408 |  | −0.027 | 0.473 |
| Cheese | −0.016 | 0.682 |  | 0.102** | 0.007 |  | 0.053 | 0.159 |  | 0.141** | <0.001 |
| Yogurt | 0.029 | 0.438 |  | −0.003 | 0.933 |  | −0.005 | 0.895 |  | 0.014 | 0.704 |
| Alcohol | 0.055 | 0.143 |  | 0.127** | <0.001 |  | 0.019 | 0.621 |  | 0.083* | 0.029 |

AEBQ, Adult Eating Behavior Questionnaire.

n=698; **p* < 0.05; ***p* < 0.01

**^a^** For *Enjoyment of food*, *r* values refer to Spearman correlations because this variable was not normally distributed.
