# Supplementary material for: Eating behaviors, dietary patterns and weight status in emerging adulthood and longitudinal associations with eating behaviors in early childhood
Source: Int J Behav Nutr Phys Act. 2022 Nov 16;19:139. doi: 10.1186/s12966-022-01376-z (PMC9670577; doi:10.1186/s12966-022-01376-z)
Supplement: Supplementary file 3 — Additional file 3: Supplementary Table 3. Bivariate (Pearson's) correlations between food avoidance scales of AEBQ and consumption of various food groups. [file 12966_2022_1376_MOESM3_ESM.docx]

**Supplementary Table 3** Bivariate (Pearson’s) correlations between food avoidance scales of AEBQ and consumption of various food groups

|  | Satiety responsiveness | |  | Emotional undereating | |  | Food fussiness^a^ | |  | Slowness in eating | |
| --- | --- | --- | --- | --- | --- | --- | --- | --- | --- | --- | --- |
|  | Corr | *P* value |  | Corr | *P* value |  | Corr | *P* value |  | Corr | *P* value |
| Sugar-sweetened beverages | 0.048 | 0.208 |  | 0.001 | 0.973 |  | 0.149** | <0.001 |  | 0.055 | 0.148 |
| Fatty/salty snacks & French fries | −0.029 | 0.438 |  | −0.044 | 0.245 |  | 0.064 | 0.090 |  | 0.052 | 0.172 |
| Sweet snacks & desserts | −0.066 | 0.083 |  | −0.036 | 0.338 |  | 0.167** | <0.001 |  | −0.073 | 0.054 |
| Fruit | −0.022 | 0.553 |  | −0.002 | 0.957 |  | −0.254** | <0.001 |  | −0.021 | 0.582 |
| Juice | 0.033 | 0.387 |  | −0.014 | 0.705 |  | −0.088* | 0.020 |  | 0.025 | 0.512 |
| Vegetables | −0.030 | 0.429 |  | −0.049 | 0.199 |  | −0.324** | <0.001 |  | 0.006 | 0.877 |
| Non-whole-grain products | −0.093* | 0.014 |  | −0.042 | 0.266 |  | 0.150** | <0.001 |  | −0.055 | 0.148 |
| Whole-grain products | −0.088* | 0.020 |  | −0.078* | 0.039 |  | −0.197** | <0.001 |  | 0.023 | 0.544 |
| Processed meat, pizza & fried  chicken/fish/shellfish | −0.117** | 0.002 |  | −0.048 | 0.201 |  | 0.151** | <0.001 |  | −0.048 | 0.205 |
| Red meat | −0.062 | 0.104 |  | −0.074 | 0.051 |  | 0.087* | 0.022 |  | −0.049 | 0.200 |
| Poultry, fish, shellfish (excluding fried) & eggs | −0.145** | <0.001 |  | −0.036 | 0.346 |  | −0.121** | 0.001 |  | −0.062 | 0.100 |
| Legumes, nuts & seeds | −0.074 | 0.052 |  | −0.090* | 0.017 |  | −0.281** | <0.001 |  | −0.001 | 0.970 |
| Milk & plant-based drinks (unsweetened) | 0.001 | 0.985 |  | 0.000 | 0.996 |  | −0.039 | 0.303 |  | 0.032 | 0.401 |
| Cheese | −0.066 | 0.080 |  | −0.001 | 0.972 |  | −0.047 | 0.219 |  | 0.018 | 0.644 |
| Yogurt | −0.028 | 0.460 |  | −0.017 | 0.661 |  | −0.092* | 0.015 |  | 0.076* | 0.043 |
| Alcohol | 0.036 | 0.338 |  | 0.029 | 0.442 |  | −0.226** | <0.001 |  | −0.055 | 0.150 |

AEBQ, Adult Eating Behavior Questionnaire.

n=698; **p* < 0.05; ***p* < 0.01

**^a^** For *Food fussiness*, *r* values refer to Spearman correlations because this variable was not normally distributed.
