# Supplementary material for: Eating behaviors, dietary patterns and weight status in emerging adulthood and longitudinal associations with eating behaviors in early childhood
Source: Int J Behav Nutr Phys Act. 2022 Nov 16;19:139. doi: 10.1186/s12966-022-01376-z (PMC9670577; doi:10.1186/s12966-022-01376-z)
Supplement: Supplementary file 4 — Additional file 4: Supplementary Table 4. Bivariate associations between AEBQ scales and dietary patterns at age 22 years. [file 12966_2022_1376_MOESM4_ESM.docx]

**Supplementary Table 4** Bivariate associations between AEBQ scales and dietary patterns at age 22 years

| Dietary pattern |  | | | | |
| --- | --- | --- | --- | --- | --- |
| AEBQ scale | ß | (SE) | *P* value | R^2^ | R^2^_adj_ |
| Healthy dietary pattern |  |  |  |  |  |
| Hunger | 0.108* | (0.044) | 0.014 | 0.009 | 0.007 |
| Food responsiveness | 0.025 | (0.044) | 0.574 | 0.001 | −0.001 |
| Emotional overeating | 0.049 | (0.033) | 0.141 | 0.003 | 0.002 |
| Enjoyment of food | 0.164** | (0.048) | 0.001 | 0.017 | 0.015 |
| Satiety responsiveness | −0.071 | (0.041) | 0.086 | 0.004 | 0.003 |
| Emotional undereating | −0.058 | (0.032) | 0.075 | 0.005 | 0.003 |
| Food fussiness | −0.355** | (0.035) | <0.001 | 0.128 | 0.127 |
| Slowness in eating | 0.019 | (0.032) | 0.544 | 0.001 | −0.001 |
| Beverage-rich dietary pattern |  |  |  |  |  |
| Hunger | −0.004 | (0.039) | 0.918 | 0.000 | −0.001 |
| Food responsiveness | −0.055 | (0.039) | 0.156 | 0.003 | 0.001 |
| Emotional overeating | −0.029 | (0.029) | 0.327 | 0.001 | −0.000 |
| Enjoyment of food | −0.167** | (0.042) | <0.001 | 0.022 | 0.021 |
| Satiety responsiveness | −0.028 | (0.036) | 0.445 | 0.001 | −0.001 |
| Emotional undereating | −0.026 | (0.028) | 0.363 | 0.001 | −0.000 |
| Food fussiness | 0.205** | (0.032) | <0.001 | 0.055 | 0.054 |
| Slowness in eating | 0.028 | (0.028) | 0.314 | 0.001 | 0.000 |
| Protein-rich dietary pattern |  |  |  |  |  |
| Hunger | −0.034 | (0.038) | 0.359 | 0.001 | −0.000 |
| Food responsiveness | −0.053 | (0.038) | 0.164 | 0.003 | 0.001 |
| Emotional overeating | −0.053 | (0.028) | 0.061 | 0.005 | 0.004 |
| Enjoyment of food | 0.084* | (0.041) | 0.043 | 0.006 | 0.004 |
| Satiety responsiveness | −0.105** | (0.035) | 0.003 | 0.013 | 0.011 |
| Emotional undereating | −0.044 | (0.028) | 0.115 | 0.004 | 0.002 |
| Food fussiness | −0.027 | (0.032) | 0.402 | 0.001 | −0.000 |
| Slowness in eating | −0.043 | (0.027) | 0.113 | 0.004 | 0.002 |
| High energy density dietary pattern |  |  |  |  |  |
| Hunger | 0.008 | (0.038) | 0.842 | 0.000 | −0.001 |
| Food responsiveness | 0.050 | (0.038) | 0.188 | 0.002 | 0.001 |
| Emotional overeating | −0.007 | (0.029) | 0.808 | 0.000 | −0.001 |
| Enjoyment of food | −0.003 | (0.042) | 0.942 | 0.000 | −0.001 |
| Satiety responsiveness | −0.100** | (0.035) | 0.006 | 0.011 | 0.010 |
| Emotional undereating | −0.035 | (0.028) | 0.208 | 0.002 | 0.001 |
| Food fussiness | 0.106** | (0.032) | 0.002 | 0.015 | 0.014 |
| Slowness in eating | −0.026 | (0.027) | 0.340 | 0.001 | −0.000 |

AEBQ, Adult Eating Behavior Questionnaire. R^2^_adj_, Adjusted R squared.

**p* < 0.05; ***p* < 0.01

Based on simple linear regressions testing whether appetitive traits are associated with dietary patterns (n=698).
