# Supplementary material for: Eating behaviors, dietary patterns and weight status in emerging adulthood and longitudinal associations with eating behaviors in early childhood
Source: Int J Behav Nutr Phys Act. 2022 Nov 16;19:139. doi: 10.1186/s12966-022-01376-z (PMC9670577; doi:10.1186/s12966-022-01376-z)
Supplement: Supplementary file 5 — Additional file 5: Supplementary Table 5. Bivariate associations between eating behaviors in early childhood and eating behaviors/patterns at age 22 years. [file 12966_2022_1376_MOESM5_ESM.docx]

**Supplementary Table 5** Bivariate associations between eating behaviors in early childhood and eating behaviors/patterns at age 22 years

|  | Fussy eating in early childhood | | | | |  | Overeating in early childhood | | | | |
| --- | --- | --- | --- | --- | --- | --- | --- | --- | --- | --- | --- |
|  | ß | (SE) | *P* value | *R^2^* | *R^2^_adj_* |  | ß | (SE) | *P* value | *R^2^* | *R^2^_adj_* |
| **AEBQ scale** |  |  |  |  |  |  |  |  |  |  |  |
| Hunger | 0.01 | (0.02) | 0.65 | 0.000 | −0.001 |  | −0.02 | (0.03) | 0.48 | 0.001 | −0.001 |
| Food responsiveness | 0.01 | (0.02) | 0.66 | 0.000 | −0.001 |  | 0.02 | (0.03) | 0.49 | 0.001 | −0.001 |
| Emotional overeating | 0.03 | (0.03) | 0.30 | 0.002 | 0.000 |  | 0.05 | (0.04) | 0.21 | 0.002 | 0.001 |
| Enjoyment of food | −0.02 | (0.02) | 0.31 | 0.002 | 0.000 |  | 0.02 | (0.03) | 0.42 | 0.001 | −0.001 |
| Satiety responsiveness | 0.03 | (0.02) | 0.22 | 0.002 | 0.001 |  | −0.07* | (0.03) | 0.03 | 0.010 | 0.010 |
| Emotional undereating | 0.06* | (0.03) | 0.04 | 0.010 | 0.010 |  | −0.06 | (0.04) | 0.14 | 0.003 | 0.002 |
| Food fussiness | 0.14** | (0.02) | <0.001 | 0.050 | 0.050 |  | −0.03 | (0.04) | 0.33 | 0.001 | −0.000 |
| Slowness in eating | 0.05 | (0.03) | 0.06 | 0.010 | 0.004 |  | −0.15** | (0.04) | <0.001 | 0.020 | 0.020 |
| **Dietary pattern** |  |  |  |  |  |  |  |  |  |  |  |
| Healthy | −0.05 | (0.02) | 0.05 | 0.010 | 0.004 |  | −0.02 | (0.04) | 0.66 | 0.000 | −0.001 |
| Beverage-rich | 0.03 | (0.02) | 0.15 | 0.003 | 0.002 |  | 0.07* | (0.03) | 0.03 | 0.010 | 0.010 |
| Protein-rich | −0.02 | (0.02) | 0.32 | 0.001 | 0.000 |  | 0.04 | (0.03) | 0.24 | 0.002 | 0.001 |
| High energy density | 0.02 | (0.02) | 0.44 | 0.001 | −0.001 |  | 0.01 | (0.03) | 0.79 | 0.000 | −0.001 |

AEBQ, Adult Eating Behavior Questionnaire; R^2^_adj_, Adjusted R squared.

**p* < 0.05; ***p* < 0.01

Based on simple linear regressions testing whether eating behaviors in early childhood are predictors of appetitive traits or dietary patterns at age 22 years (n=698).
