# Supplementary material for: Eating behaviors, dietary patterns and weight status in emerging adulthood and longitudinal associations with eating behaviors in early childhood
Source: Int J Behav Nutr Phys Act. 2022 Nov 16;19:139. doi: 10.1186/s12966-022-01376-z (PMC9670577; doi:10.1186/s12966-022-01376-z)
Supplement: Supplementary file 6 — Additional file 6: Supplementary Table 6. Associations between eating behaviors in early childhood and weight status at age 22 years. [file 12966_2022_1376_MOESM6_ESM.docx]

**Supplementary Table 6** Associations between eating behaviors in early childhood and weight status at age 22 years

|  | Unadjusted | | |  | Adjusted^a^ | | | | |
| --- | --- | --- | --- | --- | --- | --- | --- | --- | --- |
| Behavior in early childhood | OR | 95% CI | *P* value |  | OR | 95% CI | *P* value | *R^2^* | *R^2^_adj_* |
| Fussy eating | 0.95 | 0.86; 1.06 | 0.354 |  | 0.97 | 0.87; 1.07 | 0.515 | 0.035 | 0.033 |
| Overeating | 1.48 | 1.26; 1.73 | <0.001 |  | 1.44 | 1.23;1.70 | <0.001 | 0.049 | 0.047 |

BMI, Body mass index; R^2^_adj_, Adjusted R squared.

Based on ordinal logistic regression testing whether eating behaviors in early childhood are predictors of weight status at age 22 years (n=696). BMI was calculated as kg/m^2^ based on self-reported height and weight - corrected [1]. Outcome variable comprises three categories: underweight/normal weight (BMI≤ 24.9); overweight (BMI 25.0-29.9); obese (BMI ≥30).

^a^ Analyses adjusted for sex and both maternal education and family income when QLSCD participants were children. Values for *R^2^* refer toMcFadden’s pseudo-R squared.

[1] Shields M, Gorber SC, Janssen I, Tremblay MS. Bias in self-reported estimates of obesity in Canadian health surveys: an update on correction equations for adults. Health Rep. 2011;22:35-45.
