# Supplementary material for: Eating behaviors, dietary patterns and weight status in emerging adulthood and longitudinal associations with eating behaviors in early childhood
Source: Int J Behav Nutr Phys Act. 2022 Nov 16;19:139. doi: 10.1186/s12966-022-01376-z (PMC9670577; doi:10.1186/s12966-022-01376-z)
Supplement: Supplementary file 7 — Additional file 7: Supplementary Table 7. Significant sex interactions in longitudinal analyses and stratified effects of eating behaviors in early childhood. [file 12966_2022_1376_MOESM7_ESM.docx]

**Supplementary Table 7** Significant sex interactions in longitudinal analyses and stratified effects of eating behaviors in early childhood

| Association | Interaction (Ref Male) | | |  | Stratified effects | | | |
| --- | --- | --- | --- | --- | --- | --- | --- | --- |
|  | ß | (SE) | *P* value |  | Sex | ß | 95% CI | *P* value |
| Fussy eating (childhood) −  Food responsiveness (22 y) | −0.11** | (0.04) | 0.01 |  |  |  |  |  |
|  |  |  |  |  | Female | 0.05* | 0.00; 0.10 | 0.047 |
|  |  |  |  |  | Male | −0.06 | −0.12; 0.00 | 0.062 |
| Fussy eating (childhood) −  Emotional overeating (22 y) | −0.12* | (0.05) | 0.03 |  |  |  |  |  |
|  |  |  |  |  | Female | 0.08* | 0.01; 0.14 | 0.016 |
|  |  |  |  |  | Male | −0.04 | −0.12; 0.04 | 0.313 |
| Fussy eating (childhood) −  Enjoyment of food (22 y) | −0.09* | (0.04) | 0.02 |  |  |  |  |  |
|  |  |  |  |  | Female | −0.02 | −0.03; 0.06 | 0.492 |
|  |  |  |  |  | Male | −0.07* | −0.13; 0.02 | 0.011 |
| Overeating (childhood) −  Protein-Rich pattern (22 y) | −0.13* | (0.06) | 0.03 |  |  |  |  |  |
|  |  |  |  |  | Female | 0.06 | −0.01; 0.14 | 0.085 |
|  |  |  |  |  | Male | −0.07 | −0.16; 0.02 | 0.144 |

**p* < 0.05, ***p* < 0.01
